# Clinical Evaluation of Commercial Atlas-Based Auto-Segmentation in the Head and Neck Region

**DOI:** 10.3389/fonc.2019.00239

**Published:** 2019-04-09

**Authors:** Hyothaek Lee, Eungman Lee, Nalee Kim, Joo ho Kim, Kwangwoo Park, Ho Lee, Jaehee Chun, Jae-ik Shin, Jee Suk Chang, Jin Sung Kim

**Affiliations:** Department of Radiation Oncology, Yonsei Cancer Center, Yonsei University College of Medicine, Seoul, South Korea

**Keywords:** auto-contouring, contouring, segmentation, atlas segmentation, atlas-based auto-segmentation

## Abstract

**Background:** While atlas segmentation (AS) has proven to be a time-saving and promising method for radiation therapy contouring, optimal methods for its use have not been well-established. Therefore, we investigated the relationship between the size of the atlas patient population and the atlas segmentation auto contouring (AC) performance.

**Methods:** A total of 110 patients' head planning CT images were selected. The mandible and thyroid were selected for this study. The mandibles and thyroids of the patient population were carefully segmented by two skilled clinicians. Of the 110 patients, 100 random patients were registered to 5 different atlas libraries as atlas patients, in groups of 20 to 100, with increments of 20. AS was conducted for each of the remaining 10 patients, either by simultaneous atlas segmentation (SAS) or independent atlas segmentation (IAS). The AS duration of each target patient was recorded. To validate the accuracy of the generated contours, auto contours were compared to manually generated contours (MC) using a volume-overlap-dependent metric, Dice Similarity Coefficient (DSC), and a distance-dependent metric, Hausdorff Distance (HD).

**Results:** In both organs, as the population increased from *n* = 20 to *n* = 60, the results showed better convergence. Generally, independent cases produced better performance than simultaneous cases. For the mandible, the best performance was achieved by *n* = 60 [DSC = 0.92 (0.01) and HD = 6.73 (1.31) mm] and the worst by *n* = 100 [DSC = 0.90 (0.03) and HD = 10.10 (6.52) mm] atlas libraries. Similar results were achieved with the thyroid; the best performance was achieved by *n* = 60 [DSC = 0.79 (0.06) and HD = 10.17 (2.89) mm] and the worst by *n* = 100 [DSC = 0.72 (0.13) and HD = 12.88 (3.94) mm] atlas libraries. Both IAS and SAS showed similar results. Manual contouring of the mandible and thyroid required an average of 1,044 (±170.15) seconds, while AS required an average of 46.4 (±2.8) seconds.

**Conclusions:** The performance of AS AC generally increased as the population of the atlas library increased. However, the performance does not drastically vary in the larger atlas libraries in contrast to the logic that bigger atlas library should lead to better results. In fact, the results do not vary significantly toward the larger atlas library. It is necessary for the institutions to independently research the optimal number of subjects.

## Introduction

Manual contouring of target and critical structures is resource intensive aspect of the radiotherapy planning process. In order to reduce time and workload imposed on the clinicians and operators, multiple reports have suggested the use of atlas-based automatic segmentation (1–6). Atlas segmentation involves the process of aligning the target patient (TP) to the “template” patient through “template alignment” for the contours available within the atlas library. The next step is “contour alignment” where the selected atlas patient's (AP) contours are aligned with the anatomical structures of the TP. Once the contours are aligned to the anatomical structures of TP, these contours will undergo “label fusion” process, where deformation of the contours of selected AP is performed to match the anatomical structure of the TP. This overall process of atlas segmentation enables automatic segmentation for OAR and target contouring with considerable accuracy. However, in most cases, authors have suggested manual correction before practical use. Furthermore, to our knowledge, there have been no studies regarding the optimal number of patients needed to populate an atlas library for auto-segmentation, or regarding a reasonable explanation for patient characteristics.

In this study, we focused on evaluating the optimal number of atlas patients (AP) required by the atlas library to automatically generate accurate segmentation volume for the mandible and thyroid in head and neck cancer treatment. In addition, we also evaluated whether atlas-segmentation resulted in significant time-savings, based on the number of patients and on each constituent process.

## Methods

### Patient Selection

All patients selected for this study were with a brain tumor or head and neck cancer who underwent head CT scanning. Several exclusion criterions were considered; patients below age 20, patients who underwent surgical resection, patients without thyroid and patients with a bite block or tracheostomy tube during simulation were excluded from the patient group.

According to the criterions mentioned above, a total of 110 head and neck cancer patients at Yonsei Cancer Center were randomly selected and divided into two groups (Table 1): (a) 10 target patients (TPs) to be auto segmented by “Atlas Segmentation” with commercially available software, MIM Maestro 6.7 (MIM Software Inc., Cleveland, OH); and (b) 100 atlas patients (APs) were registered to MIM as atlas atlas patients to guide performance of atlas segmentation. The study protocol conformed to the ethical guidelines of the 1975 Declaration of Helsinki, as revised in 1983, and was approved by institutional review board (IRB) of Yonsei University Health System without the IRB number. The patient records/information were anonymized and de-identified prior to analysis, and informed consent was not obtained from each participants.

**Table 1:** Patient characteristics in this study.

	**Male**	**Female**	**Average age**	**Dental artifact**	**No teeth**	**CT with open mouth**
Target patients (10)	7	3	56.3	8	1	0
Atlas patients (100)	68	32	53.9	77	10	6

**Figure 1:**
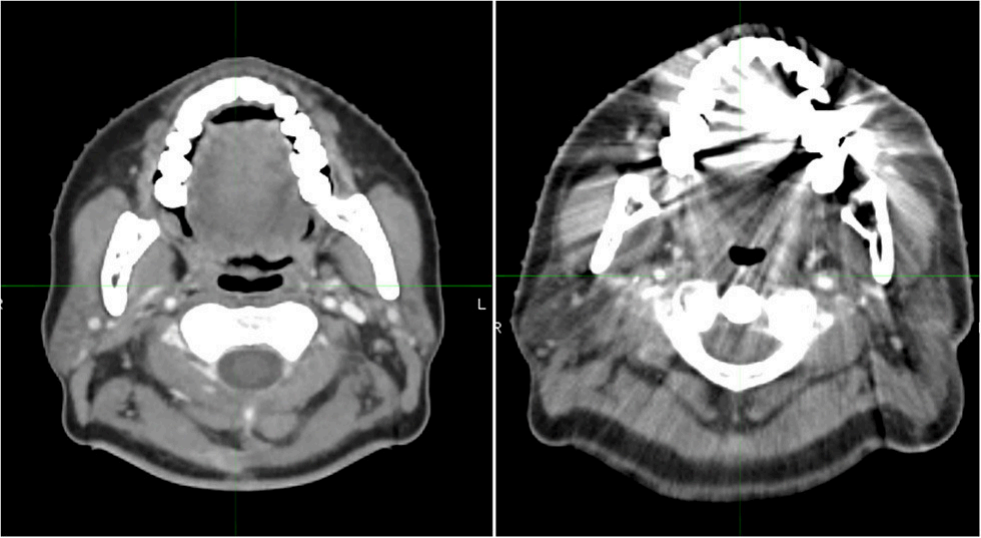
Images of two patients with **(left)** and without **(right)** dental artifacts.

All TPs were adults (age range: 33–77 years old) with brain cancer. The slice thickness of CT images was 3 mm; all CT images were taken with a closed mouth, using different angles of head inclination. Seven of 10 TPs were male and three were female. Eight of ten TPs exhibited a dental artifact Figure 1.

Of 100 APs, 68 were male and 32 were female. The average age of the AP group was 53.9 years old (range: 19–91 years old). Of 100 APs, 77 patients' CT images displayed a dental artifact within 1–3 slices; six patients' CT images were taken with an open mouth. The slice thickness of CT images was 3 mm for all APs.

Manual segmentation of the mandible and thyroid for all 110 patients involved in this study was performed by two experienced clinicians. Manually segmented contours (MCs) of 10 TPs were used as the gold standard for comparison with the MIM Atlas Segmentation-generated contours (ACs) for precision and accuracy analysis. Automatic segmentation of the mandible and thyroid of 10 TPs was conducted with the “Atlas Segmentation” function in MIM Maestro 6.7 software. Five different atlas libraries, containing *n* = 20, 40, 60, 80, and 100 APs were created to observe the effect of the population of atlas APs on the accuracy of AC.

We chose mandible and thyroid in the head and neck region, as these require the most laborious work during manual contouring. Although mandible might be considered as comparably simple organ to draw owing to its rigid shape, this applies if dental artifacts are not present. As proven by the random selection of patients, in this study, majority had dental artifacts which caused severe difficulties in manual contouring. Even in other OARs, each of their characteristics and the following difficulties need to be considered during manual contour process. Hence it is necessary to conduct a preliminary test, for whichever organ it may be, before clinical use of atlas-based auto segmentation.

### MIM Atlas Library Build Up

Five atlas libraries were created to contain a population of APs of size *n*: *n* = 20, 40, 60, 80, and 100. For every AP, planning CT images and respective manual contours of mandible and thyroid were registered to each atlas library. The entire registration process was conducted by the author to reduce inter-observer variability during the Template-AP alignment procedure and the AP-TP alignment process during “Atlas Segmentation.” It should be noted that for growing the number of n for each atlas library (e.g., from an atlas library of *n* = 20 to *n* = 40), the previous group's atlas APs were kept, and an additional 20 patients were included to generate a new atlas library.

### Atlas Segmentation

**Figure 2:**
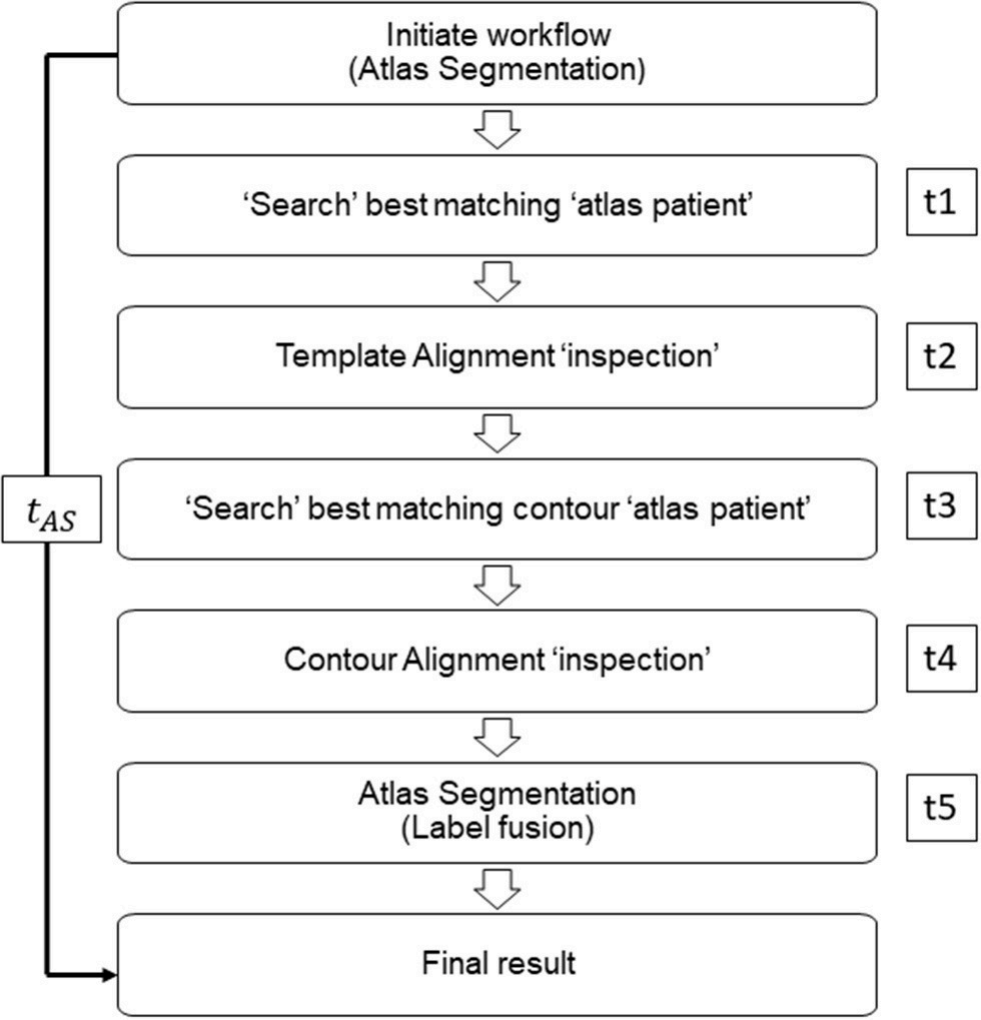
Representative workflow image of MIM atlas segmentation process. Times t1, t3, and t5 are defined as MIM time; times t2 and t4 are defined as operator time.

ACs were generated for all TPs with each atlas library (*n* = 20, 40, 60, 80, and 100). During “Template Alignment” process, TP is aligned with the “template” patient of the atlas library to be assessed for the available contours within the atlas library. The “template” patient is used as a reference for choosing an appropriate atlas subject when the operator is trying to segment a new TP. Other patients within the atlas library are aligned with the “template” patient such that they share the same reference. Each atlas library will have its own “template” patient, and it is recommended that an average shaped patient of the population within the atlas library is selected as the “template” patient. It is desirable to avoid a really heavy or skinny person or patient with any uncommon neck or mouth angle. The “Contour Alignment” process involves manual alignment of selected AP's contours to the anatomical region of interest of TP. This process involves further registration of a specific AP to the TP which helps fine-tune the alignment. It is to be noted that for this study both “Template Alignment” and “Contour Alignment” processes were conducted manually due to decreased accuracy to the resulting AC with automatic alignment. When the alignment of template and AP with the TP is manually set, the best fit atlas patients (AP) were automatically selected and the contours were automatically deformed onto the TP's CT scan. The “label fusion” process is conducted which involves the deformation of the original AP contours into the best contour shape matching to the TP anatomy (7–9). (Figure 2 describes the steps of Atlas Segmentation of MIM). This process describes the deformation of the contours from each AP to the TP. The combination of multiple contours from several AP is done by this “label fusion” process to form a single, final contour on the TP either using one of the two label fusion algorithms. The author would like to denote that there are two label fusion algorithms supported by MIM; the Majority Vote (MV) algorithm and the Simultaneous Truth and Performance Level Estimation (STAPLE) method. In MV method, votes are counted for each propagated label, and the label with most votes will be selected to generate the final segmentation (10). STAPLE, estimates the performance parameters and a probabilistic estimate of the true segmentation, by iterated estimation according to the expectation maximization algorithm (11). In this study, MV method was used throughout this study to maintain consistency.

### Simultaneous and Independent Cases

**Figure 3:**
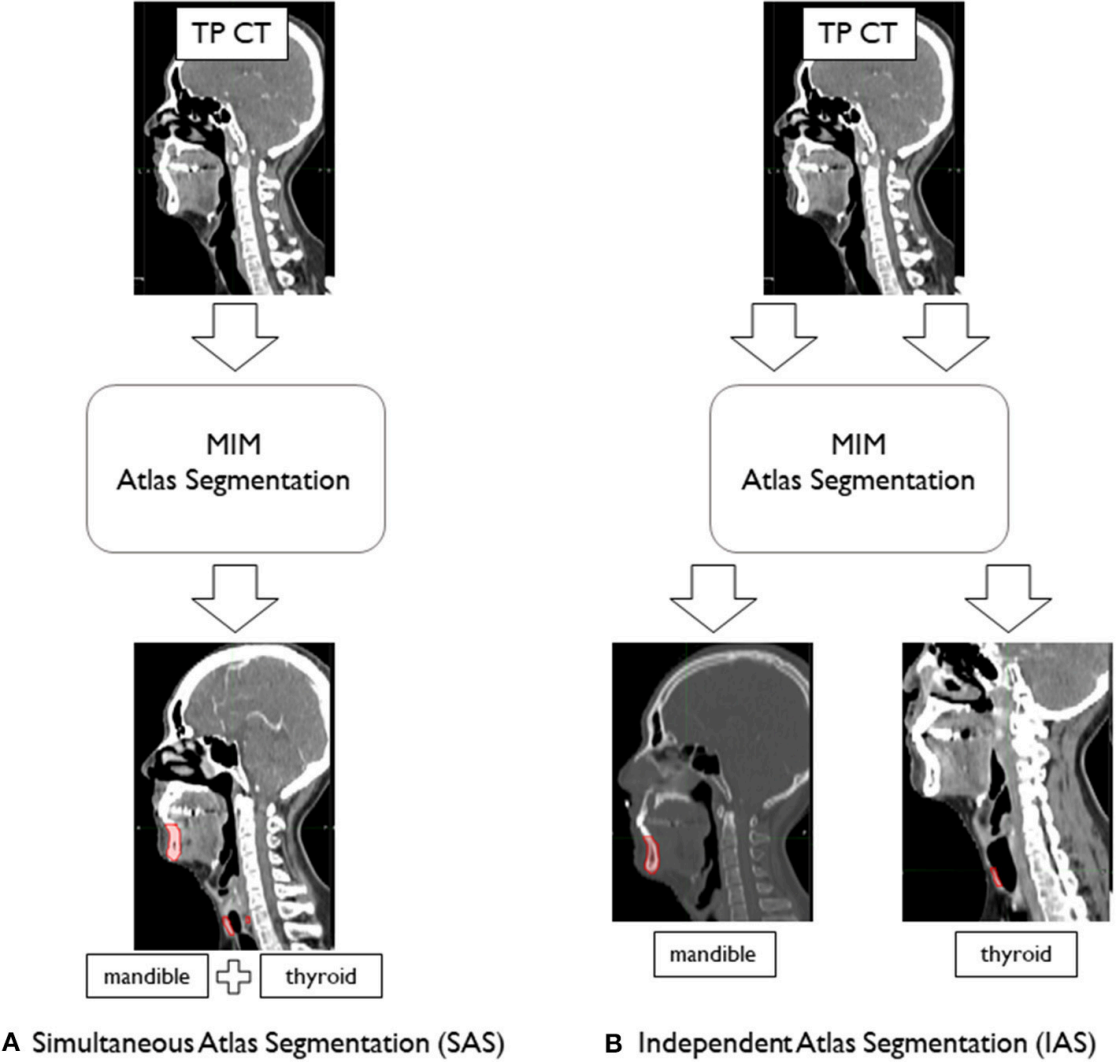
Atlas segmentation of mandible and thyroid of target patients (TP) using MIM to generate auto-segmentation; **(A)** simultaneous atlas segmentation and **(B)** independent atlas segmentation.

We performed two different atlas segmentations for mandible and thyroid. In the first, simultaneous atlas segmentation (SAS), mandible and thyroid were both included as subject organs to be auto-segmented and were generated by a single “Atlas Segmentation” process. To be specific, the contour alignment process as explained in the earlier section, involved global alignment of two contours; mandible and thyroids at the same time. In this case, the mismatch of mandible and thyroids due to head inclination was neglected. To reduce this problem of mismatching caused by head inclination difference between patients, independent atlas segmentation (IAS) was performed and each organ was independently segmented by running the “Atlas Segmentation” process twice (Figure 3). In IAS, the contour alignment process will involve alignment of individual contour and hence needs to be performed multiple times depending on the number of AC to generate. The results were recorded to analyze the difference between simultaneous and independent cases.

### Time Stamp for Atlas-Based Segmentation

The time to produce AC (*t*_*AC*_) for each TP for all atlas libraries was recorded and compared with manual segmentation time (*t*_*MC*_). Previous studies verified significant time-saving occurred when atlas-based auto-segmentation was compared with manual segmentation. Together with the standard comparison, in this study, we aimed to record the differences in time between atlas libraries. It should be noted that for this study, only MIM time (t1, t3, t5) was considered for comparison, as the operator time (t2, t4) was inconsistent between TPs and atlas libraries (Figure 2). We tested the significance between AC generation times for different atlas libraries by using Kruskal–Wallis and Mann–Whitney non-parametric statistical tests.

### Validation Method

AC of mandible and thyroid were compared with the MC produced by the clinicians. Dice Similarity Coefficient (DSC) and Hausdorff Distance (HD) metrics were used for accuracy analysis (12, 13). DSC is an overlap-based metric that measures the degree of overlap between two different volumes and is defined by the equation:

$$DSC=\;\frac{2(V_{AC}\cap V_{MC})}{V_{AC}+V_{MC}}$$

where *V*_*AC*_ is the atlas-based auto-segmentation volume of the organs of interest and *V*_*MC*_ is the manual segmentation volume. The value of DSC is expressed on a scale with a range of (0, 1); it is an effective method for evaluating the overlap of the AC with the gold standard MC. However, as suggested by Kim et al. (14), DSC may be ambiguous regarding local discrepancies.

In contrast, HD considers distance differences between two surfaces, thereby eliminating the ambiguity of the volume-wise DSC metric. Given two point sets A = {*a*_1_, …, *a*_*p*_} and B = {*b*_1_, …, *b*_*q*_}, HD is defined as:

H(A,B)=max(h(A,B),h(B,A))

Where

h(A,B)=‖a−b‖ 

The function h(A, B) is designated as the *directed* HD from A to B. It identifies a point, *a* ∈ *A*, that is furthest from the nearest neighbor in B. Effectively, it describes the most mismatched distance of a point on A to B. If h(A, B) = d, then each point of A must be within distance d of a given point on B. With function H(A, B), if HD is d, then every point on A must be within distance d of a given point on B; the reverse function is also applicable.

For both simultaneous and independent cases, target patient AC, generated by each atlas library, was compared with the gold standard MC, created by the clinicians. For the results of each n group, DSC values and HD values were averaged; the values were compared between different n population groups. The overlap limit for automatic image segmentation suggested by the literature is DSC > 0.75 (15). Additionally, Loi et al. (16) suggested that, for volumes > 30 ml, the suitable accepted limit should be DSC > 0.85.

## Results

**Figure 4:**
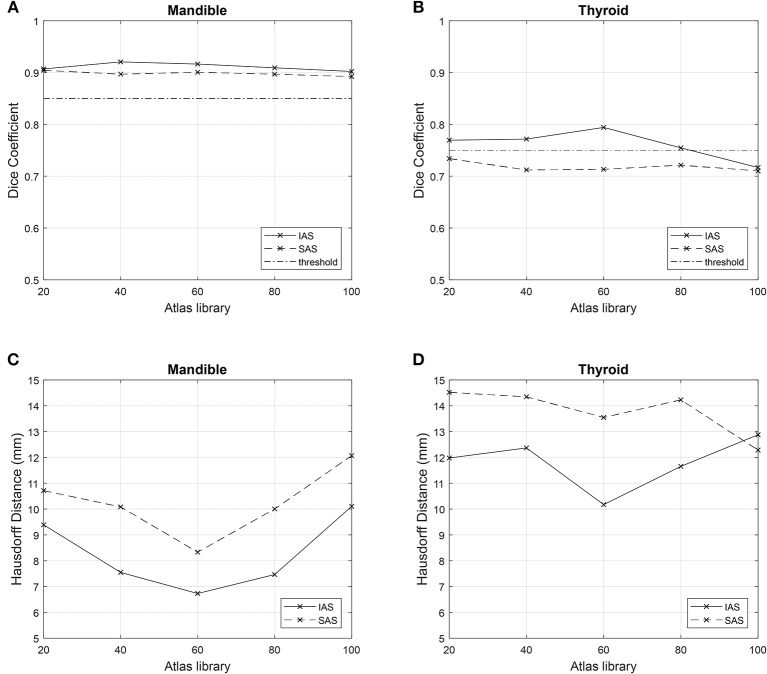
Comparison of metrics among n atlas groups. Simultaneous atlas segmentation (SAS) is represented as dashed line and independent atlas segmentation (IAS) is represented as bold line. Good overlap limit of 0.75 and 0.85 are represented as straight horizontal lines in **(A,B)**. **(A)** Mean Dice Similarity Coefficient for mandible. **(B)** Mean Dice Similarity Coefficient for thyroid. **(C)** Mean Hausdorff Distance for mandible. **(D)** Mean Hausdorff Distance for thyroid.

All 10 target patients were auto-segmented with each atlas library. Resulting ACs of mandible and thyroid were compared with the gold standard MCs to achieve mean DSC and mean HD. Mean DSC and HD values obtained from AC–MC comparisons for each library for mandible and thyroid are summarized in Table 2. For each atlas library group, the respective mean DSC values and mean HD values showed improvements when mandible and thyroid were auto-segmented using IAS method. As shown in Figure 4, IAS showed better results than SAS.

**Table 2:** Mean dice similarity coefficient and hausdorff distance values for multiple n atlas libraries.

		***n***	**20**	**40**	**60**	**80**	**100**
SAS	Mandible	DSC (sd)	0.90 (0.03)	0.90 (0.06)	0.90 (0.01)	0.90 (0.02)	0.89 (0.02)
		HD (sd)	10.72 (4.82)	10.08 (5.62)	8.33 (1.76)	10.00 (4.99)	12.07 (6.94)
	Thyroid	DSC (sd)	0.73 (0.08)	0.71 (0.16)	0.71 (0.16)	0.72 (0.16)	0.71 (0.14)
		HD (sd)	14.52 (4.33)	14.35 (4.81)	13.55 (5.56)	14.23 (5.20)	12.28 (3.06)
IAS	Mandible	DSC (sd)	0.91 (0.03)	0.92 (0.01)	0.92 (0.01)	0.91 (0.02)	0.90 (0.03)
		HD (sd)	9.39 (4.47)	7.55 (2.00)	6.73 (1.31)	7.46 (1.69)	10.10 (6.52)
	Thyroid	DSC (sd)	0.77 (0.08)	0.77 (0.07)	0.79 (0.06)	0.75 (0.07)	0.72 (0.13)
		HD (sd)	11.98 (2.28)	12.37 (2.66)	10.17 (2.89)	11.65 (3.73)	12.88 (3.94)

### SAS

For mandible, the *n* = 60 atlas library produced the best result: DSC = 0.90 (0.01) and HD = 8.33 (1.76) mm. However, for thyroid, the best result was produced by the *n* = 20 atlas library; DSC = 0.73 (0.08) and HD = 14.52 (4.33) mm. Worst results were produced by the *n* = 100 atlas library for the mandible, where DSC = 0.89 (0.02) and HD = 12.07 (6.94) mm. For thyroid, the worst result was shown by the *n* = 40 atlas library; DSC = 0.71 (0.16) and HD = 14.35 (4.81) mm (Table 2).

### IAS

For both mandible and thyroid, the best results were produced by the *n* = 60 atlas library. For mandible, the values were: DSC = 0.92 (0.01) and HD = 6.73 (1.31) mm. For thyroid, the values were: DSC = 0.79 (0.06) and HD = 10.17 (2.89) mm. The worst results were produced by *n* = 100 atlas library for both mandible and thyroid, where DSC = 0.90 (0.03) and HD = 10.10 (6.52) mm for the mandible, and DSC = 0.72 (0.13) and HD = 12.33 (3.94) mm for thyroid (Table 2).

### Time Stamp

The shortest mean time was achieved by *n* = 20 groups for IAS mandible, IAS thyroid, and SAS with 40.1 ± 2.65 s, 42.3 ± 3.16 s, and 46.4 ± 2.85 s respectively. Although the scale of time increase can be considered negligible, mean time to generate AC increased as the population of the atlas library increased. For *n* = 100 atlas library, for IAS mandible, IAS thyroid and SAS, the mean time was 45.8 ± 2.45 s, 46.1 ± 1.20 s, and 49.6 ± 2.19 s respectively. When tested for significance in SAS, a statistically significant time difference occurred (*p* = 0.006) when the atlas library increased from *n* = 60 to *n* = 80. However, no statistically significant differences occurred between *n* = 20, 40, and 60, and *n* = 80 and 100.

### Anatomical Mismatches

**Figure 5:**
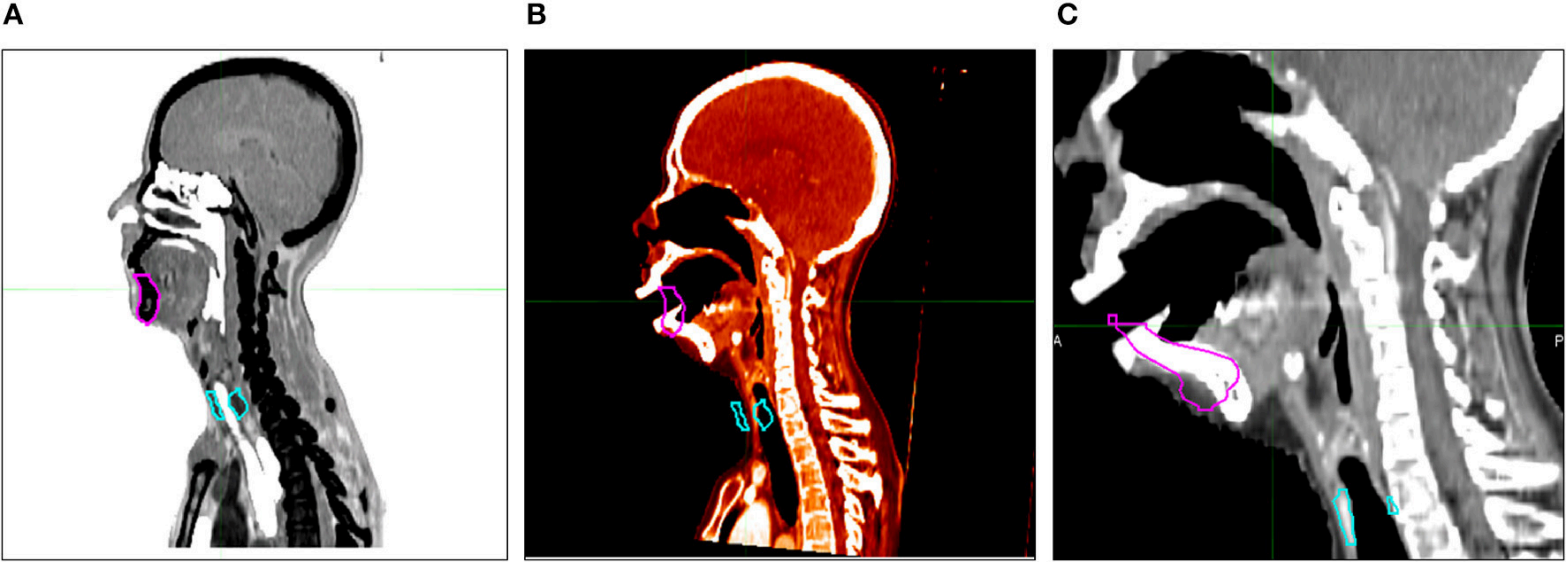
Auto-segmented contour results with open mouth target patient. **(A)** Atlas patient CT and its contour. **(B)** Target patient (open mouth) CT and selected atlas patient's contour. **(C)** Atlas segmentation-generated contour (AC).

Anatomical mismatches between AP and TP resulted in poor metric values. Regarding TPs specifically, generally poor mandible metric values were observed for patient no. 3 (SAS: DSC = 0.86 and HD = 13.65 and IAS: DSC = 0.89 and HD = 7.81 mm) due to the absence of teeth (Table 3). A separate atlas-segmentation was conducted for open-mouth TP; who's CT series were originally a part of *n* = 100 atlas library. Selected AP was of closed-mouth AP. Mismatch of mandible shapes resulted in poor AC generation (Figure 5).

**Table 3:** Mandible metrics for *n* = 80 atlas-generated atlas-based auto-segmentation: simultaneous and independent atlas segmentation (SAS and IAS).

**Patient no**.		**no. 1**	**no. 2**	no. 3^*^	**no. 4**	**no. 5**	**no. 6**	**no. 7**	**no. 8**	**no. 9**	**no. 10**	**Mean (sd)**
SAS	DSC	0.91	0.91	0.86	0.90	0.89	0.90	0.86	0.92	0.92	0.89	0.90 (0.02)
	HD (mm)	7.65	9.00	13.65	7.44	9.68	7.34	22.59	8.21	4.61	9.86	10.00 (4.99)
IAS	DSC	0.93	0.91	0.89	0.90	0.89	0.93	0.93	0.91	0.90	0.89	0.91 (0.02)
	HD (mm)	6.01	6.35	7.81	8.83	10.12	6.36	5.41	6.39	7.31	10.06	7.46 (1.69)

* *Patient without teeth*.

## Discussion

In this study, we randomly selected 110 patients and created five atlas libraries with increasing numbers of patients, in increments of 20; we aimed to observe whether the performance of atlas segmentation increased with an increasing number of patients. As described in the methods section, we chose mandible and thyroid in the head and neck region, as these require the most laborious work during manual contouring. We measured the performance of atlas segmentation by using two important parameters: DSC and HD. Regarding time to generate AC, there was no significant time difference (*p* > 0.05) when library size was increased from *n* = 60 to *n* = 100.The mean time increased until *n* = 80 for all cases, with 48.5, 49.3, and 50.1 s for IAS mandible, IAS thyroid, and SAS, respectively. However, for *n* = 100, mean time decreased to 45.8, 46.1, and 49.6 s for IAS mandible, IAS thyroid, and SAS, respectively. The decrease in mean time to generate AC from *n* = 80 to *n* = 100 needs further study with larger atlas library; i.e., *n* = 120, 140, and 160 to discuss the relationship between AC generation time and the size of the atlas library. It seems the important factor influencing the performance of the AS is not the number of patients in the atlas, but the involvement of various anatomical features. In this sense, as shown by tests with open-mouth TP, failure to choose the appropriate AP illustrates the additional requirement for creating a different atlas library to perform atlas segmentation for different patients with different anatomies or postures. Depending on CT imaging region and different postures, atlas segmentation performance can face limited application if these various factors are not included in the subject library. These results are novel and could have great implications and influence the direction of future studies regarding AC. Future studies are required to compare the resulting AC for different atlas libraries containing different postures. i.e., open mouth vs. closed mouth.

As mentioned previously, a good overlap limit has been suggested by Loi et al. For the thyroid, which is considered to be a small-volume organ (volume <30 ml), a suitable threshold value of DSC = 0.75 has been suggested. Moreover, for the mandible, which is substantially larger in volume, DSC = 0.85 threshold has been suggested as a suitable threshold. As described previously, the difference between SAS and IAS arise from the method of contour alignment. SAS create AC based on the global alignment of multiple contours between TP and AP, while IAS generate AC by comparing individual contours separately, and needs to be performed multiple times depending on the total number of organs. As shown in Figure 4, for both IAS and SAS, mandible showed good overlap results with all atlas libraries. For thyroid, IAS satisfied the overlap limit of 0.75 for all atlas library groups, except for *n* = 100. Regarding SAS, every group failed to reach the threshold of 0.75 for thyroid.

It is important to note that the overall shape of patient CT images plays a critical role in AC generation. Such shapes could include the degree of inclination of the head when CT images were taken, as well as irregularities of organ shapes between patients. Owing to these differences between patients, the resulting AC greatly depended on the contours of the selected AP. This is supported by our data, in which IAS results were generally better than those produced by SAS. In this study, for SAS, when TPs were aligned with selected APs, the alignment was made by matching the tip of the mandible between patients. Because of this aspect, the location of the thyroid often was substantially mismatched between TP and AP when the degree of inclination of the head was severe. Another aspect that resulted in a large mismatch between AC and MC was irregular thyroid shape. In this sense, a large AP contour volume matched with a small TP thyroid volume resulted in engulfment of the vein and artery adjacent to the thyroid. This resulted in the AC including a certain proportion of unwanted arteries and veins, leading to poor overlapping scores.

Another factor that could have influenced the results is the fact that the quality of the AP groups was disregarded. Seventy-seven of 100 APs exhibited a type of dental artifact (Figure 1), which severely decreased the quality of the database patients and could have affected the resulting AC. When looking at individual sub groups of AP libraries; 20, 40, 60, 80 and 100, the ratio of the number of patients without any dental artifacts and those with dental artifacts were 0.15, 0.18, 0.2, 0.23, and 0.23 respectively. Although these patients were selected in random order, the distribution of the ratio and the dispersion was regular without a large bias, and hence would have had small effect in the resulting AC between different atlas library groups. It is notable that the gender and dental artifact ratios were similar in both TP and AP groups; male: female, 7:3, and dental artifact: no artifact, 8:2.

Regarding TPs specifically, poor mandible metric values were observed for patient no. 3 in both IAS and SAS (Table 3). This was the only patient without any teeth in the TP group; hence, this patient demonstrated generally poor mandible metric scores. The generated AC mandible volume included teeth from the selected AP contour that were not present in the MC volume. This suggests a critical anatomical mismatch can lead to poor AC results with low metric scores for atlas segmentation. In a similar sense, six CT image series in this study were taken with the patients' mouth open. However, in no atlas segmentation process were these images selected to produce AC of the TP. As a related test, one of the open-mouth APs was removed from the atlas library (*n* = 100) and replaced with one of the original TPs. CT image series of the open-mouth patient were then used as a TP for atlas segmentation with atlas library *n* = 100. The resulting AC was generated with closed-mouth AP CT and contours, resulting in a very poor AC (Figure 5). In this respect, it could be useful to create different atlas groups categorized by different poses and shapes to produce more accurate AC volumes.

The author would also like to denote that a future study is required to validate the difference in performance when STAPLE algorithm is implemented. In the early stage of this study, with *n* = 20 atlas library, MV and STAPLE algorithms were both tested. However, we decided to stay with one algorithm due to insignificant difference in performance between STAPLE and MV algorithm when the scores were compared.

Our initial question regarding the optimal number of APs in an atlas library stays unclear. We expected the results to converge to better metric values as n increased; however, as shown in Figure 4, this did not occur. In contrary to the prevailing notion, our results showed that it is not necessarily true to have better results with increasing number of AP in the atlas library. Unlike the increase in performance between *n* = 20 and *n* = 60, the difference in the performance does not vary drastically in the larger atlas libraries and hence it is hard to expect a huge improvement of performance in larger atlas libraries. We come up with a hypothesis that it is not necessarily the size of the atlas library which matters in the resulting contours, but it might be dependent upon the quality of the CT images of the subjects or the quality of the relevant contours. Furthermore, the optimal size of the atlas library may be different for different organs at risk depending on the data base APs. It is crucial for different institutions to carry out independent research of the optimal number of APs in atlas library when building atlas library for atlas-based auto segmentation. In this sense, the resulting ACs from the study were considered a time-saving approach to produce auto-segmentation. Furthermore, the ACs require further manual inspection and correction by experienced clinicians before actual use in treatment planning.

A validation procedure is required to determine if robust performance can be achieved with other normal organs. A similar study must be conducted with target volumes to measure performance. If these results can be validated, further studies will be necessary to determine methodologies to achieve better performance of ABAS. Additional studies are required to determine the effects of heterogeneity and variations in anatomic features of APs within the atlas library. Moreover, with the growing popularity of the application of machine learning and deep learning techniques in a wide array of fields, the quality of automatic contouring methods may undergo further development with the implementation of these new techniques (17).

## Conclusions

We successfully evaluated the commercial atlas-based segmentation for mandible and thyroid in the head and neck region, owing to the complexity and time-consuming effort involved in manual delineation. With a cohort of 110 patients, we were able to determine the optimal number of subjects for our clinic; moreover, we discovered some interesting practical points in atlas-based automatic segmentation, such as the need for individual automatic segmentation in relation to organ positions or anatomical differences. Commercial software is not optimized for individual clinics; moreover, every clinic has different concepts for OAR and target delineation. The evaluation of the optimal size of atlas library for atlas-based segmentation should thus be performed in each clinic and for each treatment site before clinical use.

## Author Contributions

Conception, design, and drafting the manuscript were performed by HyL, EL, JSC, and JSK. Data collection and interpreting were performed by NK, JhK, KP, HoL, JC, and JS. All authors read and approved the final manuscript.

### Conflict of Interest Statement

The authors declare that the research was conducted in the absence of any commercial or financial relationships that could be construed as a potential conflict of interest.

## Abbreviations

AC: atlas segmentation-generated contours
DSC: Dice Similarity Coefficient
HD: Hausdorff Distance
IAS: independent atlas segmentation
IMRT: intensity-modulated radiation therapy
MC: manually segmented contours
MV: majority vote
OAR: organ at risk
SAS: simultaneous atlas segmentation
AP: atlas patients
TP: target patients.
